# Supplementary material for: Variation in bridgmanite grain size accounts for the mid-mantle viscosity jump
Source: Nature. 2023 Jul 5;620(7975):794–9. doi: 10.1038/s41586-023-06215-0 (PMC10447242; doi:10.1038/s41586-023-06215-0)
Supplement: Supplementary file 1 — . [file 41586_2023_6215_MOESM1_ESM.pdf]

nature portfolio

https://doi.org/10.1038/s41586-023-06215-0

Supplementary information

Variation in bridgmanite grain size accounts for the mid-mantle viscosity jump

In the format provided by the authors and unedited

Supplementary Information for

Bridgmanite grain size variation accounts for the mid-mantle viscosity jump

Hongzhan Fei[1,2*], Maxim D. Ballmer[3], Ulrich Faul[4], Nicolas Walte[5], Weiwei Cao[6], Tomoo Katsura[1,7]

1. Bayerisches Geoinstitut, Universität Bayreuth, Bayreuth, Germany.
2. School of Earth Sciences, Zhejiang University, Hangzhou, China.
3. Department of Earth Sciences, University College London, London, UK.
4. Earth Atmospheric and Planetary Sciences, Massachusetts Institute of Technology, Cambridge, MA, USA.
5. Heinz Maier-Leibnitz Zentrum (MLZ), Technische Universität München, Garching, Germany.
6. Conditions Extrêmes et Matériaux: Haute Température et Irradiation (CEMHTI), Orléans, France.
7. Center for High Pressure Science and Technology Advanced Research, Beijing, China.

*corresponding author email: H.Fei (feihongzhan@zju.edu.cn)

This Supplementary Information file contains examples of BSE images of all the samples (Figures S1 – S57).

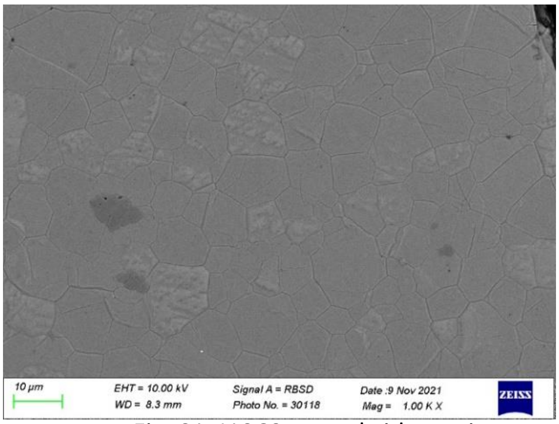

Fig. S1. I1269 – opx-bridgmanite

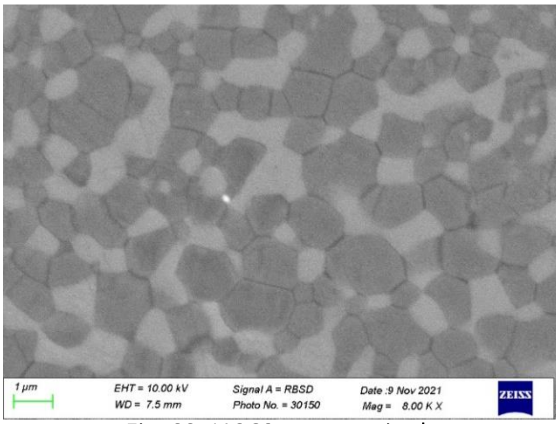

Fig. S2. I1269 – post-spinel

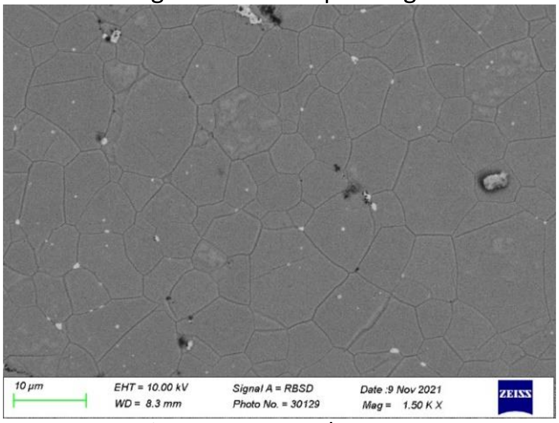

Fig. S3. I1269 – glass-1.02

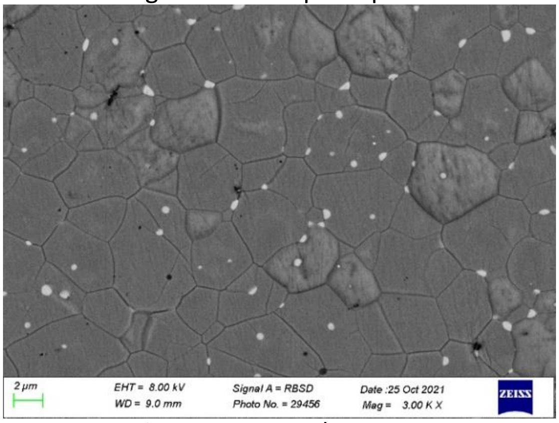

Fig. S4. I1269 – glass-1.05

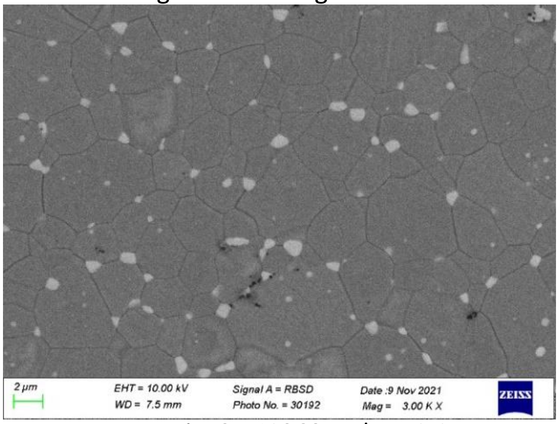

Fig. S5. I1269 – glass-1.1

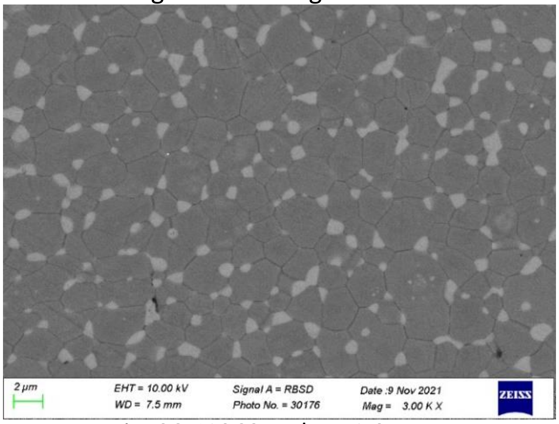

Fig. S6. I1269 – glass-1.2

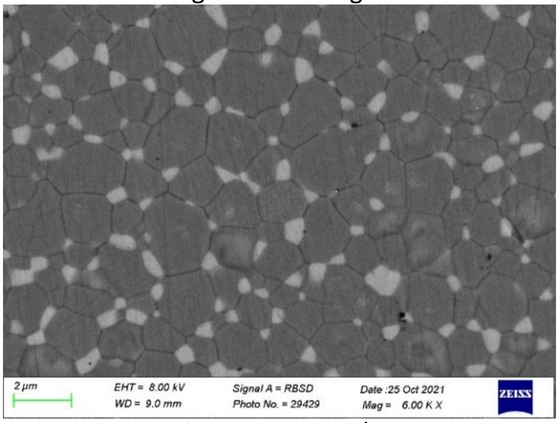

Fig. S7. I1269 – glass-1.3

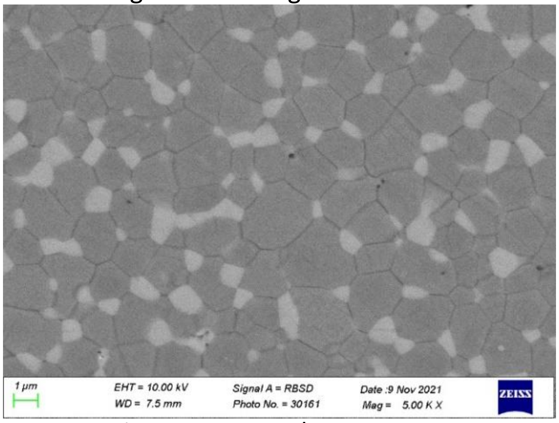

Fig. S8. I1269 – glass-1.4

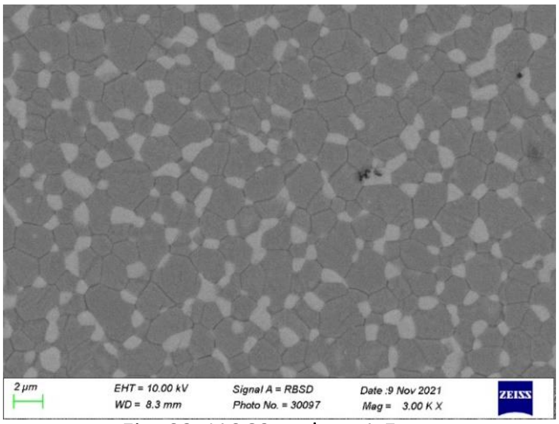

Fig. S9. I1269 – glass-1.5

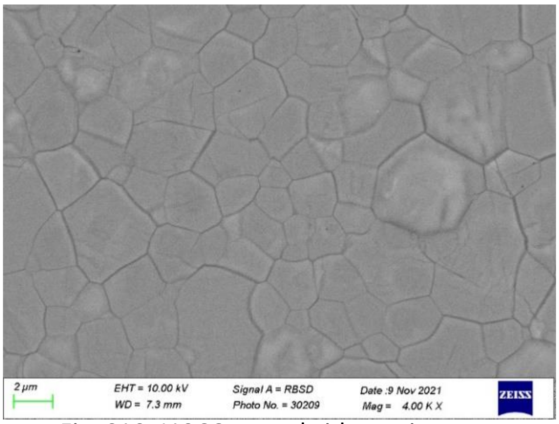

Fig. S10. I1266 – opx-bridgmanite

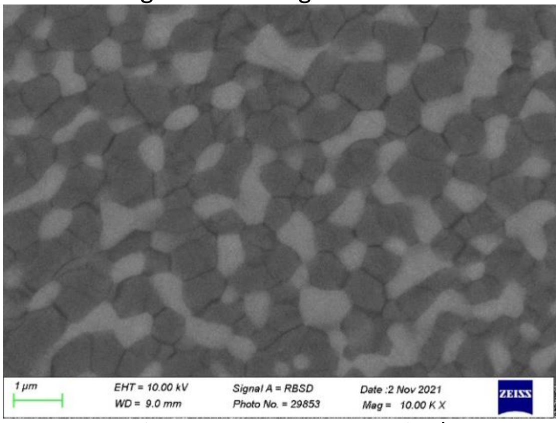

Fig. S11. I1266 – post-spinel

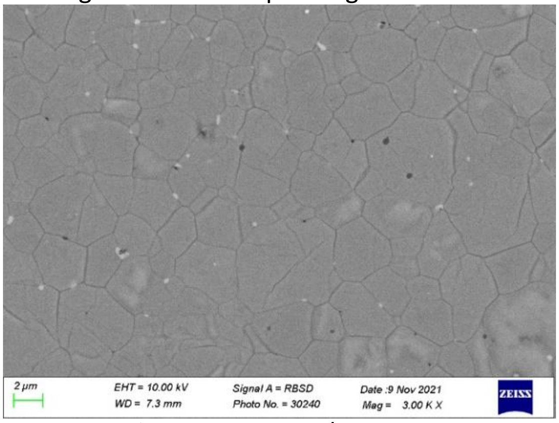

Fig. S12. I1266 – glass-1.02

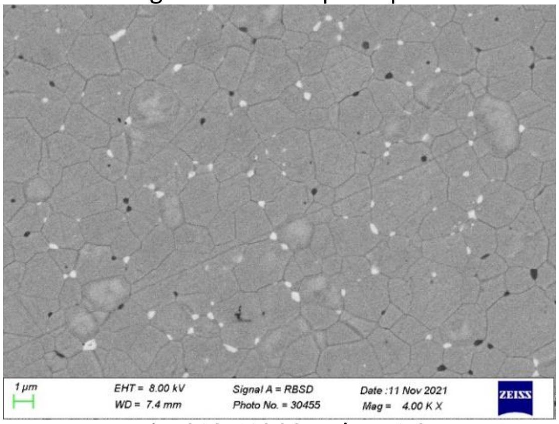

Fig. S13. I1266 – glass-1.05

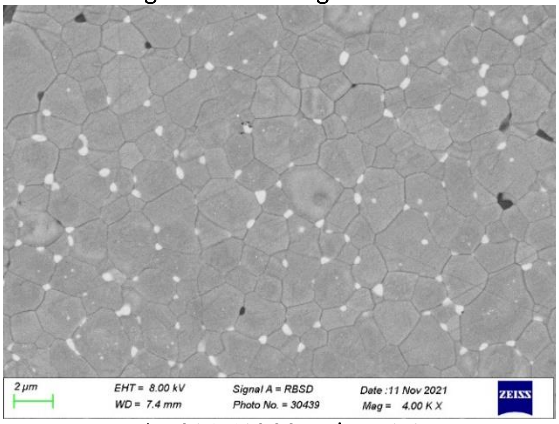

Fig. S14. I1266 – glass-1.1

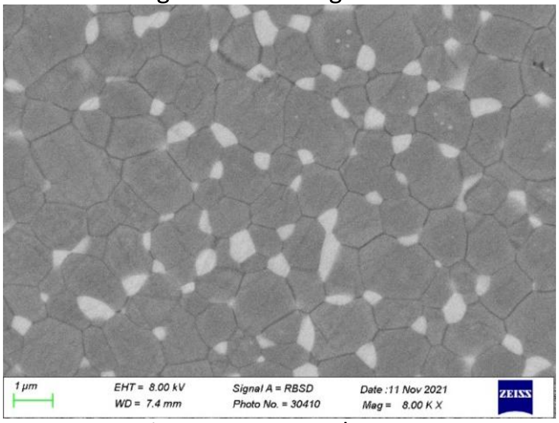

Fig. S15. I1266 – glass-1.2

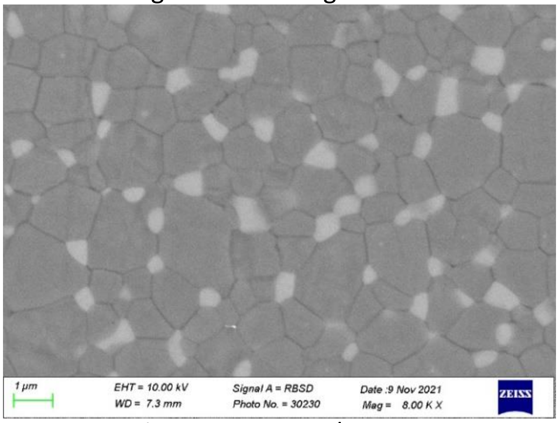

Fig. S16. I1266 – glass-1.3

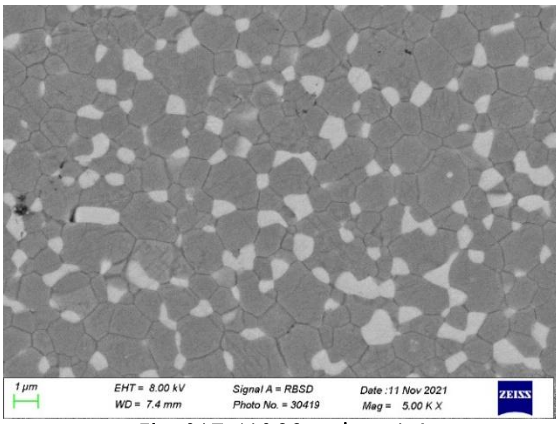

Fig. S17. I1266 – glass-1.4

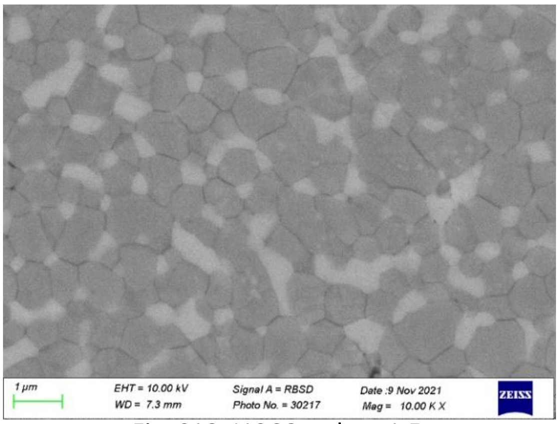

Fig. S18. I1266 – glass-1.5

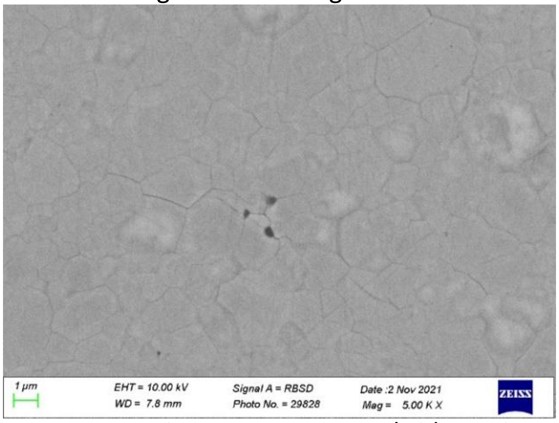

Fig. S19. I1260 – opx-bridgmanite

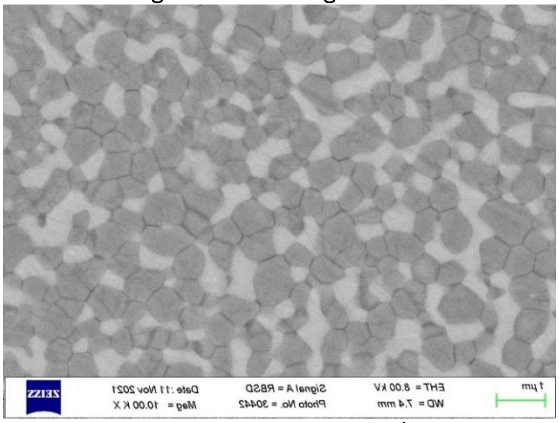

Fig. S20. I1260 – post-spinel

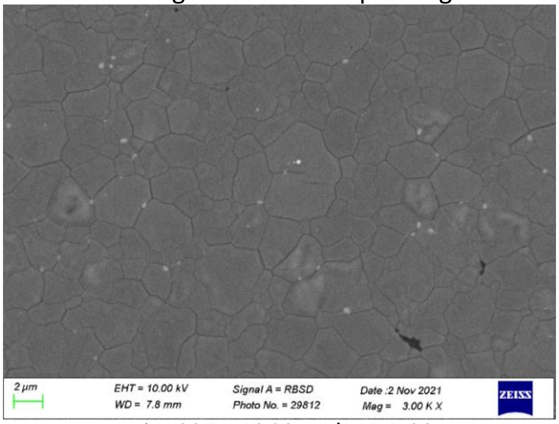

Fig. S21. I1260 – glass-1.02

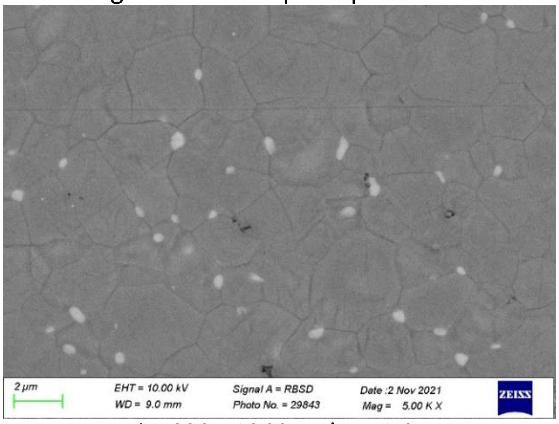

Fig. S22. I1260 – glass-1.05

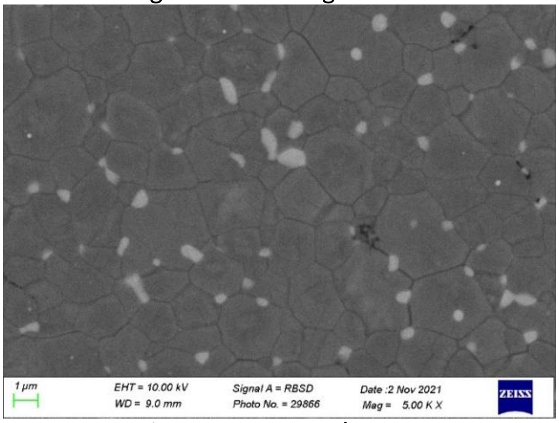

Fig. S23. I1260 – glass-1.1

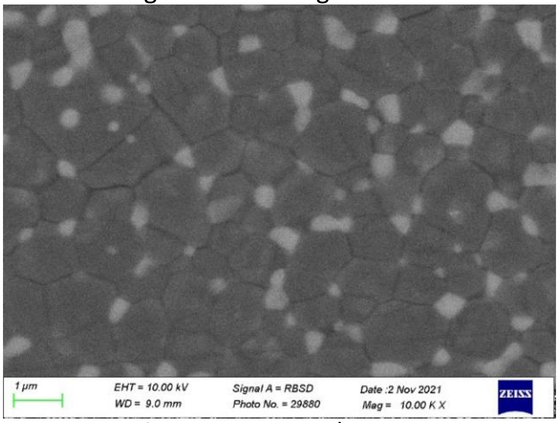

Fig. S24. I1260 – glass-1.2

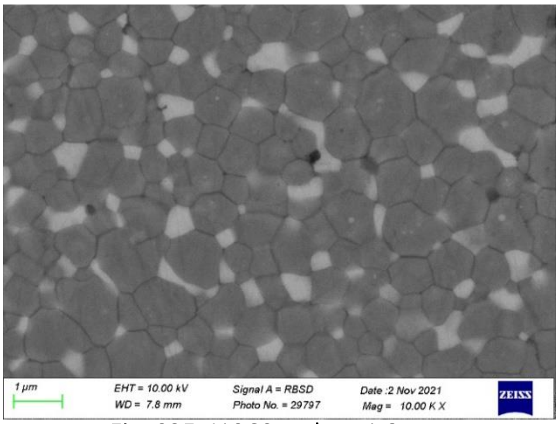

Fig. S25. I1260 – glass-1.3

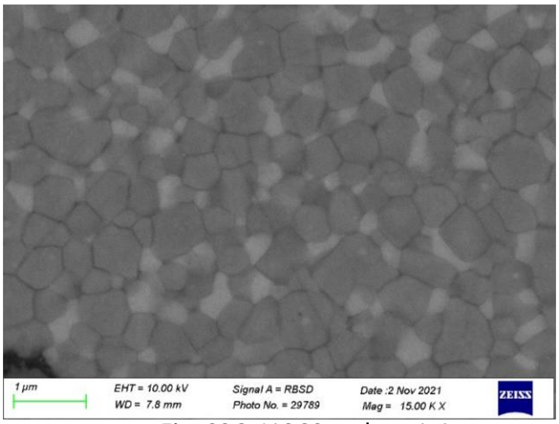

Fig. S26. I1260 – glass-1.4

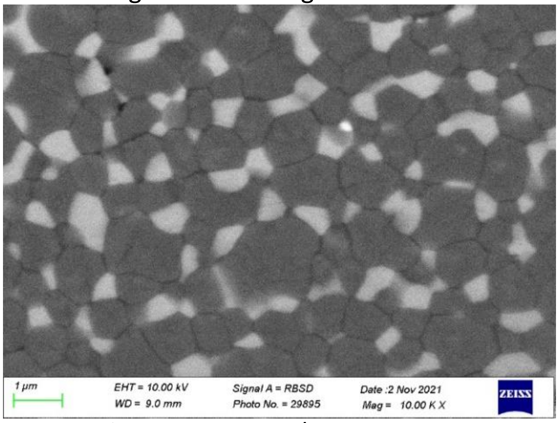

Fig. S27. I1260 – glass-1.5

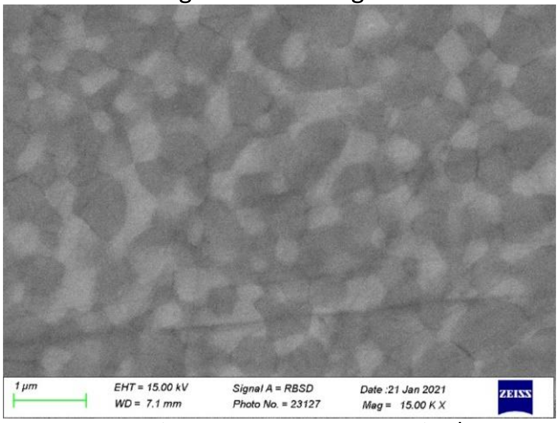

Fig. S28. I1147 – post-spinel

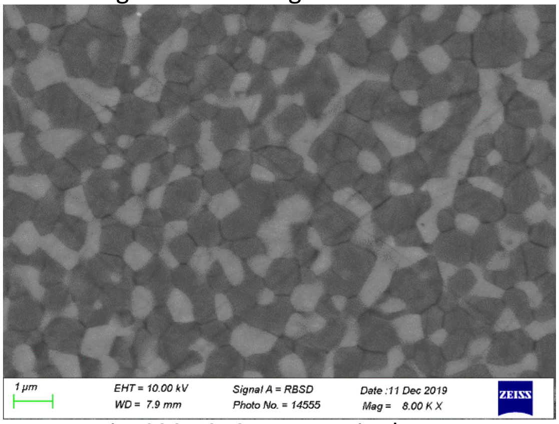

Fig. S29. I959 – post-spinel

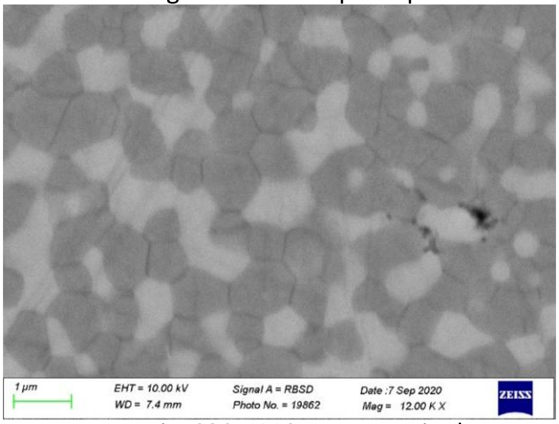

Fig. S30. I1105 – post-spinel

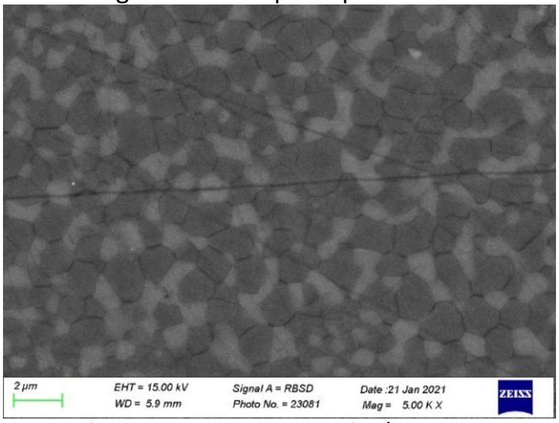

Fig. S31. I1143 – post-spinel

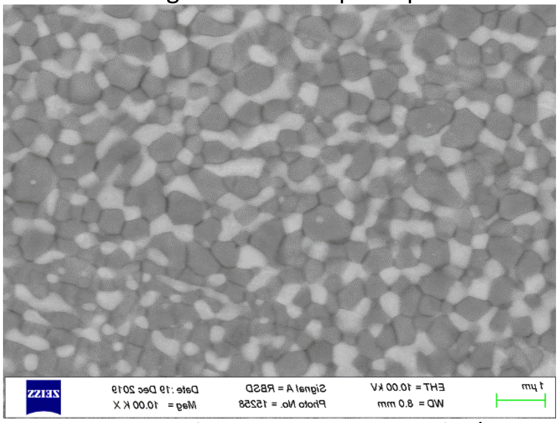

Fig. S32. I928 – post-spinel

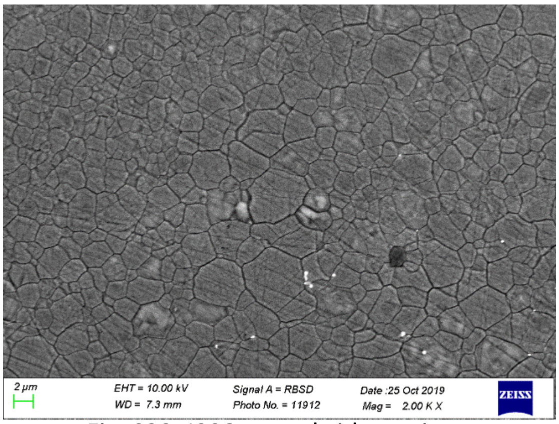

Fig. S33. I928 – opx-bridgmanite

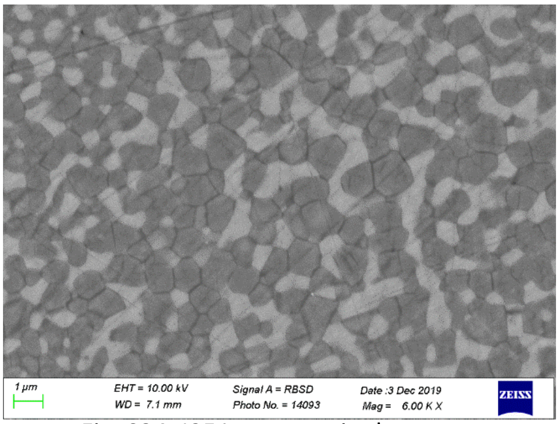

Fig. S34. I951 – post-spinel

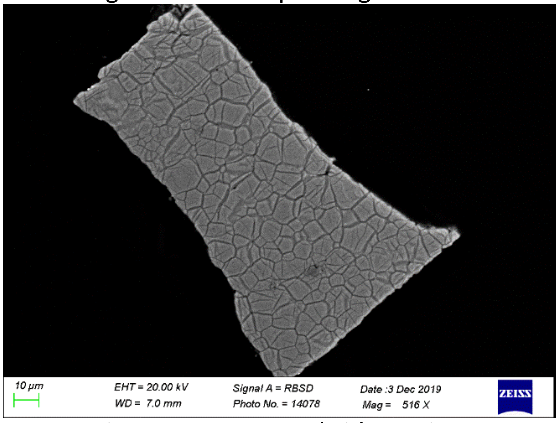

Fig. S35. I951 – opx-bridgmanite

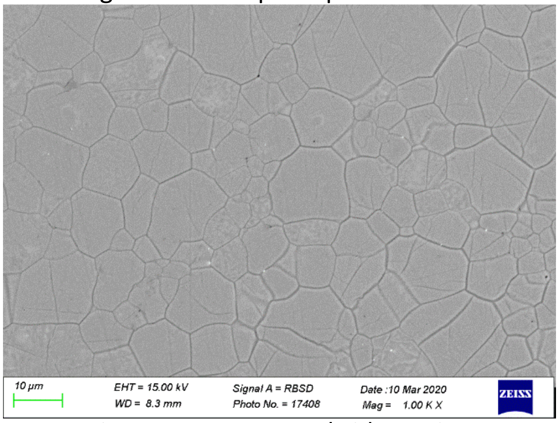

Fig. S36. I1023 – opx-bridgmanite

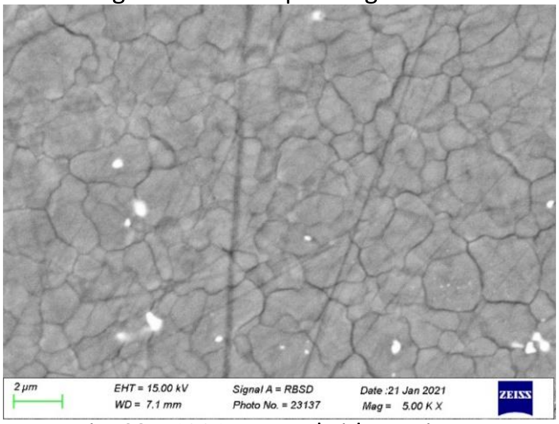

Fig. S37. I1147 – opx-bridgmanite

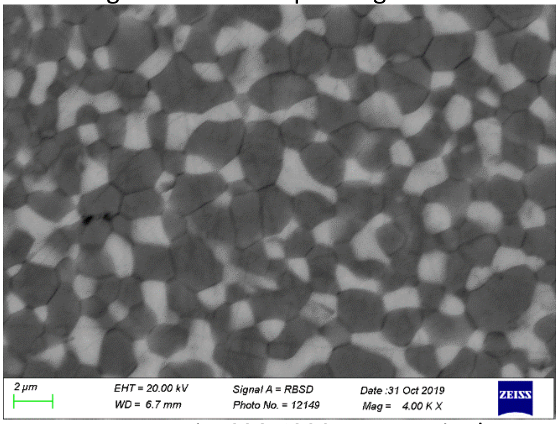

Fig. S38. I930 – post-spinel

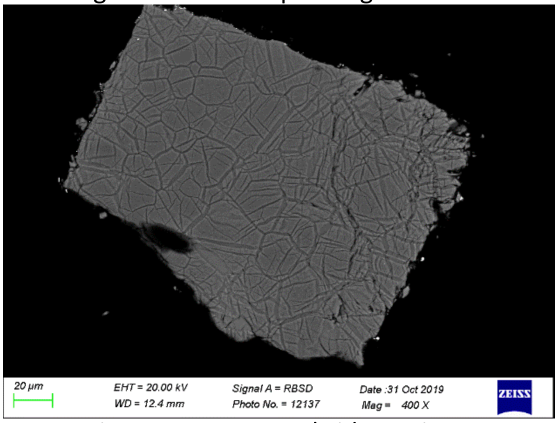

Fig. S39. I930 – opx-bridgmanite

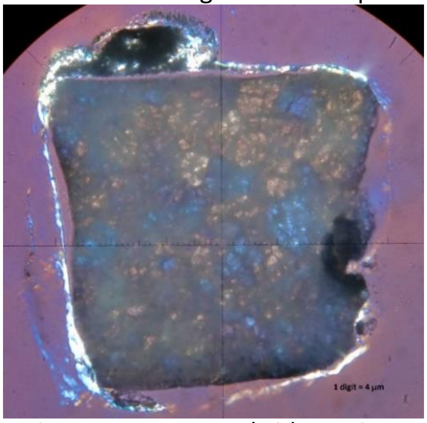

Fig. S40. I930 – opx-bridgmanite

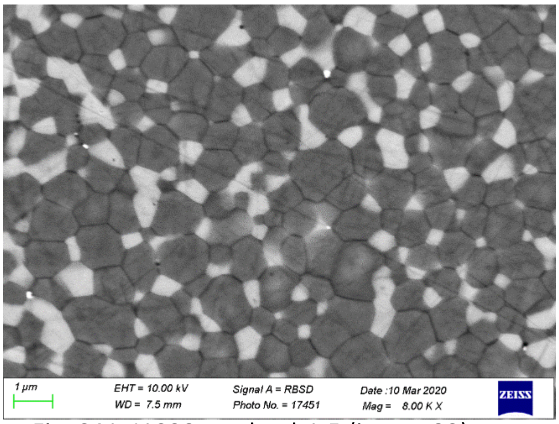

Fig. S41. I1023 – sol-gel-1.5 (image 32)

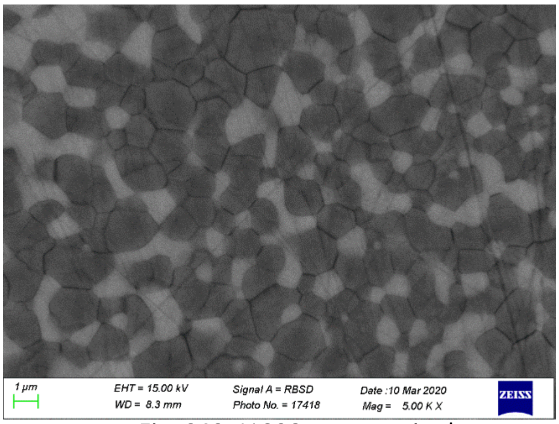

Fig. S42. I1023 – post-spinel

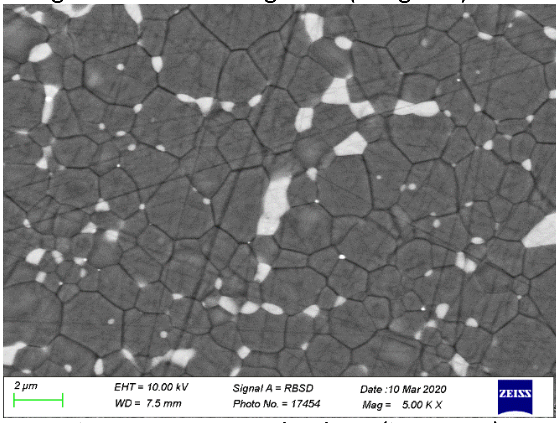

Fig. S43. I1023 – sol-gel-1.5 (image 35)

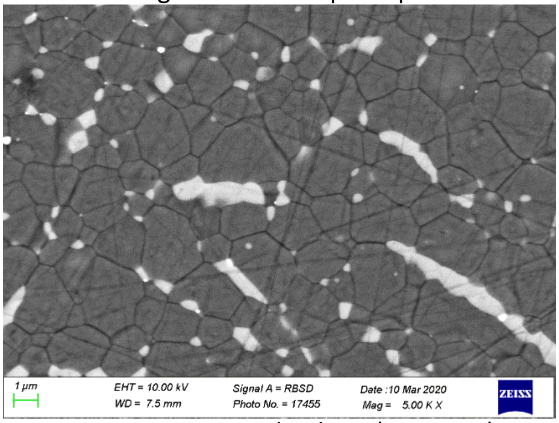

Fig. S44. I1023 – sol-gel-1.5 (image 36)

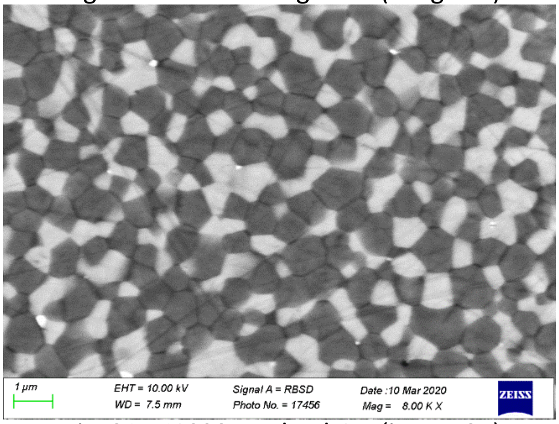

Fig. S45. I1023 – sol-gel-1.5 (image 37)

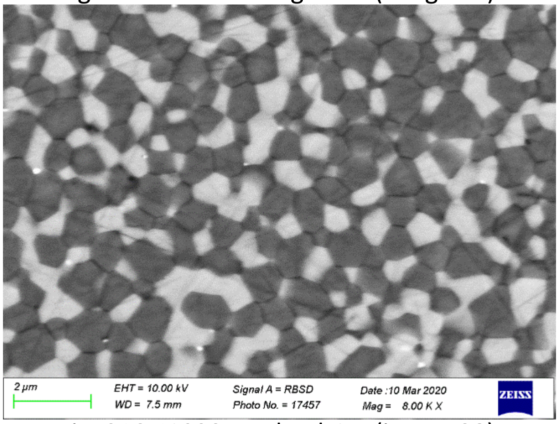

Fig. S46. I1023 – sol-gel-1.5 (image 38)

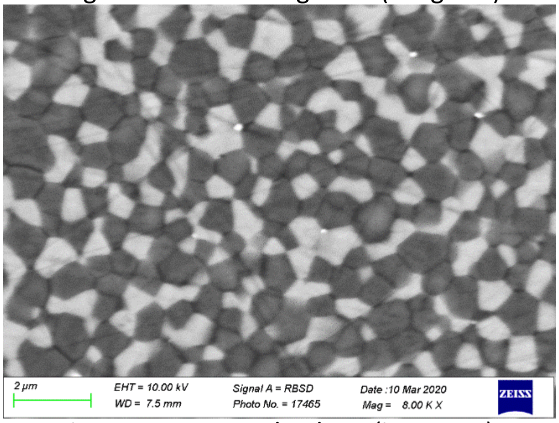

Fig. S47. I1023 – sol-gel-1.5 (image 46)

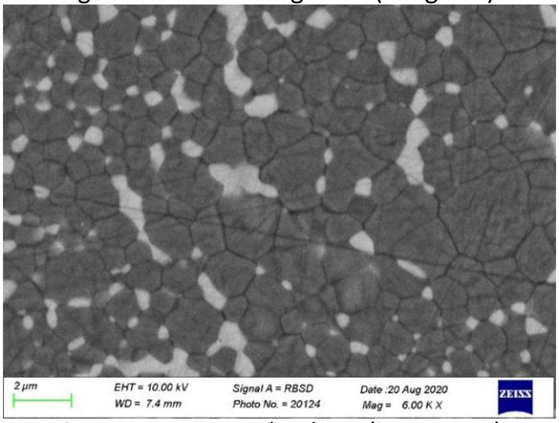

Fig. S48. I1023 – sol-gel-1.5 (image 146)

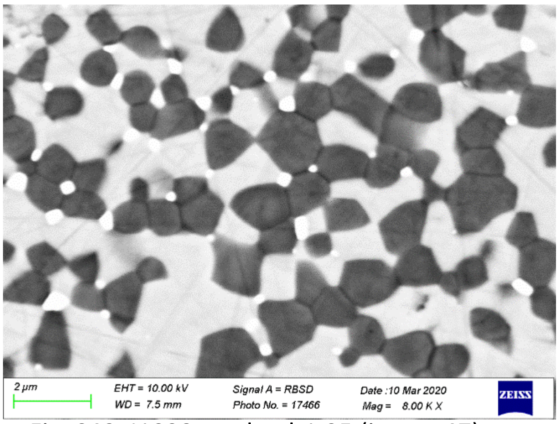

Fig. S49. I1023 – sol-gel-1.25 (image 47)

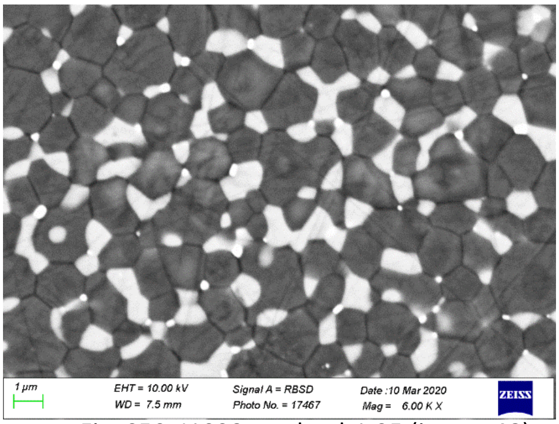

Fig. S50. I1023 – sol-gel-1.25 (image 48)

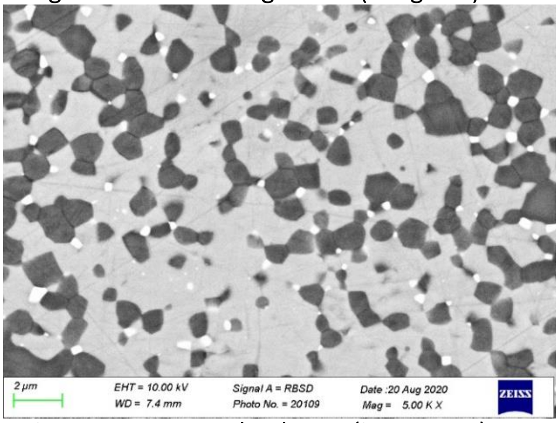

Fig. S51. I1023 – sol-gel-1.25 (image 131)

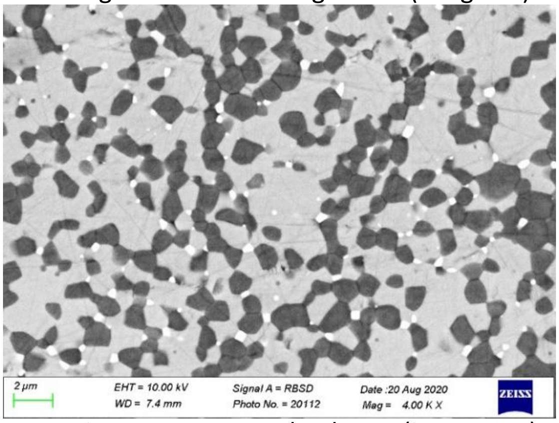

Fig. S52. I1023 – sol-gel-1.25 (image 134)

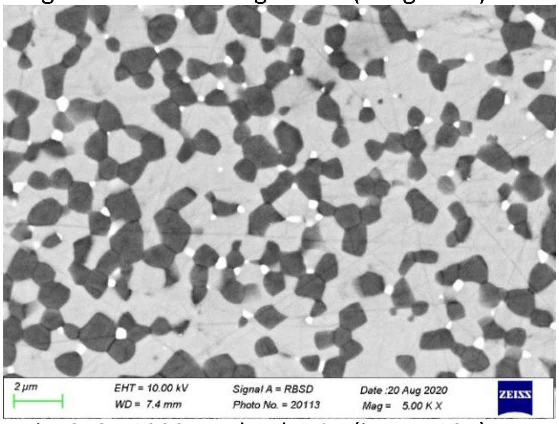

Fig. S53. I1023 – sol-gel-1.25 (image 135)

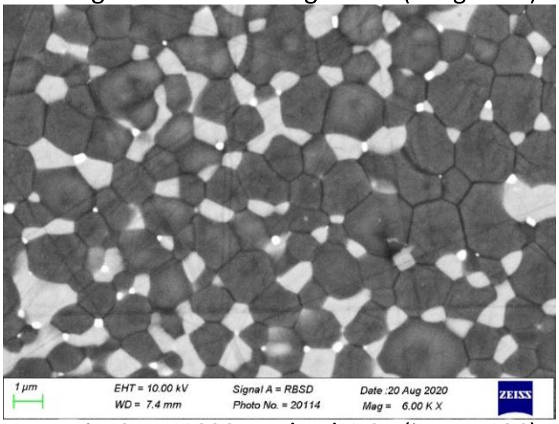

Fig. S54. I1023 – sol-gel-1.25 (image 136)

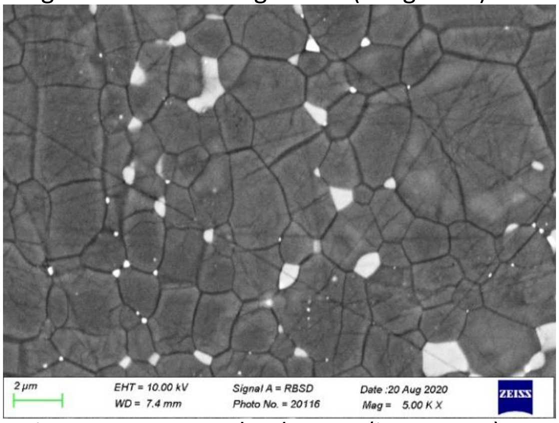

Fig. S55. I1023 – sol-gel-1.125 (image 138)

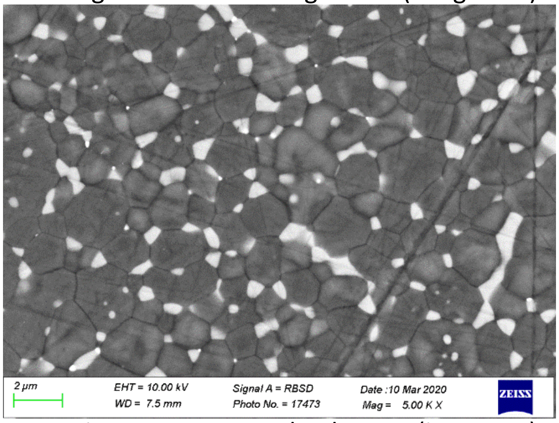

Fig. S56. I1023 – sol-gel-1.125 (image 54)

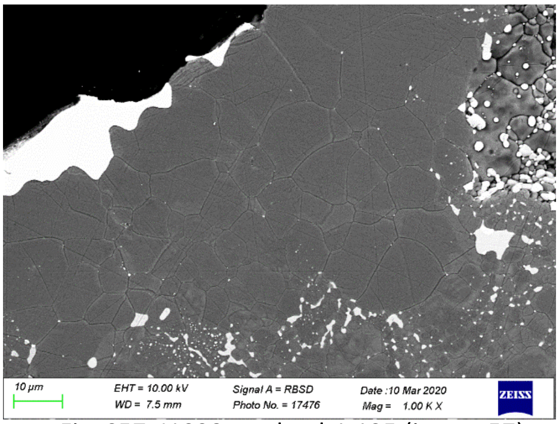

Fig. S57. I1023 – sol-gel-1.125 (image 57)
